# MicroRNA-451a and Th1/Th2 ratio inform inflammation, septic organ injury, and mortality risk in sepsis patients

**DOI:** 10.3389/fmicb.2022.947139

**Published:** 2022-08-04

**Authors:** Feng Geng, Wei Liu, Li Yu

**Affiliations:** Department of Intensive Care Unit, Tongji Medical College, The Central Hospital of Wuhan, Huazhong University of Science and Technology, Wuhan, China

**Keywords:** sepsis, miR-451a, Th1/Th2 ratio, general disease severity, mortality risk

## Abstract

**Aims:**

MicroRNA-451a (miR-451a) regulates Th1/Th2 cell differentiation, inflammation, and septic organ injury in several experiments. Therefore, the present study aimed to explore the inter-correlation of miR-451a with the Th1/Th2 ratio, and their association with inflammation, septic organ injury, and mortality risk in patients with sepsis.

**Methods:**

Consecutively, 117 patients with sepsis and 50 healthy controls (HCs) were enrolled. Peripheral blood mononuclear cell samples were collected to detect miR-451a expression and the Th1/Th2 ratio in all subjects.

**Results:**

MiR-451a (*p* < 0.001), Th1 cells (*p* = 0.014), and the Th1/Th2 ratio (*p* < 0.001) increased, while Th2 cells (*p* < 0.001) declined in patients with sepsis compared with HCs. It was of note that miR-451a was positively correlated with Th1 cells (*p* = 0.002) and the Th1/Th2 ratio (*p* = 0.001), while it was negatively related to Th2 cells (*p* = 0.005) in patients with sepsis. Meanwhile, miR-451a and the Th1/Th2 ratio correlated with most of the following indexes: TNF-α, IL-1β, IL-6, C-reactive protein, sequential organ failure assessment (SOFA) score, or Acute Physiology and Chronic Health Evaluation II (APACHE II) score (most *p* < 0.05). Moreover, miR-451a (*p* < 0.001) and the Th1/Th2 ratio (*p* = 0.001) increased in deaths compared to survivors of sepsis; further ROC curve showed both miR-451a and the Th1/Th2 ratio possessed a certain value to predict mortality of patients with sepsis. Additionally, the Th1/Th2 ratio [odds ratio (OR): 2.052, *p* = 0.005] was independently related to 28-day mortality risk from multivariate logistic regression.

**Conclusion:**

MiR-451a correlates with the Th1/Th2 ratio, and they both relate to inflammation, septic organ injury, and mortality risk in patients with sepsis.

## Introduction

Sepsis is defined as life-threatening organ dysfunction caused by a dysregulated host response to infection according to the Third International Consensus Definitions for Sepsis and Septic Shock (Singer et al., 2016). It is predicted that approximately 31.5 million cases of sepsis occur across the world, which is further responsible for 5.3 million deaths annually (Fleischmann et al., 2016). In a recent national cross-sectional study conducted in China, sepsis has accounted for nearly 20% of intensive care unit (ICU) admission and a 35.5% mortality rate within 90 days after hospitalization (Xie et al., 2020). Apart from the high mortality rate, sepsis also induces multiple complications (such as cardiomyopathy, coagulopathy, liver injury, etc.), impairing the clinical outcomes of sepsis survivors (Iba et al., 2020; L’Heureux et al., 2020; Sun et al., 2020). Besides, lacking effective pharmacological management options remains another factor contributing to the unfavorable prognosis of patients with sepsis (Thompson et al., 2019). Hence, identifying potential prognostic biomarkers is necessary to stratify patients with sepsis and to further realize individualized treatment, which may help to improve the overall management of sepsis.

MicroRNA-451a (miR-451a), as one of the recently identified microRNAs, is reported to be dysregulated in patients with sepsis, also exhibits close involvement in regulating T cell differentiation and infection-related inflammatory responses (Cheng et al., 2017; Ahmad et al., 2020; Liu et al., 2020; Wang et al., 2020). A recent genomic study has displayed that miR-451a is one of the upregulated differentially expressed genes (DEG) in septic mice (Ahmad et al., 2020). Moreover, one *in vitro* experiment exhibits that miR-451a promotes the differentiation of CD4^+^ T cells into T helper (Th) cells in systemic lupus erythematosus (SLE) (Cheng et al., 2017). Furthermore, miR-451a is reported to upregulate the inflammatory cytokines [such as interleukin (IL)-1β, IL-6, and tumor necrosis factor (TNF)-α in septic rats (Wang et al., 2020)]. While few clinical studies explore the value of miR-451a measurement in patients with sepsis.

Thus, this study aimed to explore the inter-correlation of miR-451a with the Th1/Th2 ratio, as well as their association with inflammation, septic organ injury, and mortality risk in patients with sepsis.

## Materials and methods

### Subjects

From November 2018 to March 2021, a total of 117 patients with sepsis were recruited in this study. The eligibility criteria: (1) diagnosed as sepsis according to the definition and diagnostic criteria of sepsis in 2016 (Singer et al., 2016); (2) aged between 18 and 80 years; (3) within 24 h of the symptom onset; (4) willing to provide peripheral blood (PB) samples for research analysis. The exclusion criteria: (1) accompanied by solid tumor or blood malignancies; (2) had autoimmune diseases; (3) took immunosuppressants within 6 months before enrollment; (4) pregnant or gestational patients. Additionally, 50 healthy subjects were included in the study during the same period as health controls (HCs). Ethics Committee of The Central Hospital of Wuhan, Tongji Medical College, Huazhong University of Science and Technology permitted the study, and all the participants or their families signed informed consent.

### Data recording and blood sample collection

Demographics, comorbidities, and disease features were recorded by case report form. The Acute Physiology and Chronic Health Evaluation II (APACHE II) score and the Sequential Organ Failure Assessment (SOFA) score were assessed within 24 h after admission. PB samples were collected from 117 patients with sepsis within 24 h of the symptom onset and 50 HCs after enrollment. Follow-up continued until death or 28 days after enrollment.

### Th1/Th2 determination

Within 24 h after collection, fresh PB samples from 80 patients with sepsis and 50 HCs were analyzed using flow cytometry to detect Th1 cells (%, in CD4^+^ T cells) and Th2 cells (%, in CD4^+^ T cells) with the employment of the BD Pharmingen™ Human Th1/Th2 Phenotyping Kit (BD Biosciences, San Diego, California, United States). The FACSCanto™ II (BD Biosciences, Franklin Lakes, New Jersey, United States) was used for flow cytometry. The FlowJo (Tree Star, Ashland, Oregon, United States) was applied for processing flow cytometry data. Then, the Th1/Th2 ratio was calculated as the percentage of Th1 cells divided by the percentage of Th2 cells.

### Enzyme-linked immunosorbent assay

Serum was isolated from PB samples of 117 patients with sepsis to detect inflammatory cytokines. The levels of TNF-α, IL-1β, and IL-6 were detected by Human TNF-α enzyme-linked immunosorbent assay (ELISA) Kit, Human IL-1 beta ELISA Kit, and Human IL-6 ELISA Kit (Abcam, Waltham, Massachusetts, United States), respectively. The sensitivity or minimum detectable dose of TNF-α, IL-1β, and IL-6 using those commercial ELISA kits was found to be 4.32, 1.5, and 3 pg/mL, respectively. All procedures were strictly implemented according to the product protocol.

### Reverse transcription quantitative polymerase chain reaction assay

The peripheral blood monocyte cells (PBMCs) were isolated and proposed for miR-451a measurement using reverse transcription quantitative polymerase chain reaction (RT-qPCR) in 117 patients with sepsis and 50 HCs. Briefly, the total RNA extraction was accomplished using a PureZOL RNA isolation reagent (Bio-Rad, Hercules, California, United States), and then reverse transcription was performed by the Script™ cDNA Synthesis Kit (Bio-Rad, Hercules, California, United States). Consequently, qPCR reaction was performed by the TB Green™ Fast qPCR Mix (Takara, Kusatsu, Shiga, Japan). The relative expression of miR-451a was calculated based on the 2^–ΔΔCt^ method using U6 as an internal reference. The detailed primers were designed in line with a previous study (Wang et al., 2020).

### Statistical analysis

According to data distributions, continuous data were displayed with mean ± standard deviation (SD) or median with interquartile range (IQR). Categorical data were displayed with numbers (percentage). Comparison between two groups was analyzed by the Mann–Whitney *U*-test. Correlation analysis between two variables was determined by the Spearman’s rank correlation test. Receiver-operating characteristic (ROC) analysis was used to assess the ability of variables to distinguish two groups. The SPSS software v26.1 (IBM Corporate., Armonk, New York, United States) and the GraphPad Prism software v7.01 (GraphPad Software Inc., San Diego, California, United States) were used to complete data analysis and graph plotting. Univariate logistic regression analysis was used to assess factors affecting 28-day mortality, and then all clinical features collected from the patients with sepsis were included in the multivariate logistic regression analysis with a step-forward method. *p* < 0.05 was considered an indication of a statistical significance.

## Results

### Clinical features

The mean age of patients with sepsis was 58.1 ± 12.2 years (Table 1). There were 38 (32.5%) women and 79 (67.5%) men. The primary infection site in 43 (36.7%), 34 (29.1%), 24 (20.5%), and 16 (13.7%) patients with sepsis were abdominal infection, respiratory infection, skin and soft tissue infection, and other infections. Besides, the primary organisms in 67 (57.3%), 31 (26.5%), 11 (9.4%), and 18 (15.4%) patients with sepsis were G- bacteria, G + bacteria, fungus, and other microorganisms, leaving 17 (14.5%) patients with total culture negative. The mean APACHE II and SOFA scores were 11.8 ± 5.7 and 4.7 ± 1.8, respectively. Furthermore, the detailed clinical features of the patients with sepsis are listed in Table 1.

**TABLE 1:** Clinical data of patients with sepsis.

Items	Sepsis patients (*N* = 117)
**Demographics**	
Age (years), mean ± SD	58.1 ± 12.2
Gender, n (%)	
Female	38 (32.5)
Male	79 (67.5)
BMI (kg/m^2^), mean ± SD	22.9 ± 3.8
Smoke, n (%)	45 (38.5)
Drink, n (%)	44 (37.6)
**Comorbidities**	
History of hypertension, n (%)	46 (39.3)
History of hyperlipidemia, n (%)	19 (16.2)
History of diabetes, n (%)	14 (12.0)
History of CKD, n (%)	10 (8.5)
History of CCVD, n (%)	24 (20.5)
**Disease features**	
**Primary infection site, n (%)**	
Abdominal infection	43 (36.7)
Respiratory infection	34 (29.1)
Skin and soft tissue infection	24 (20.5)
Other infections	16 (13.7)
**Primary organism, n (%)**	
G- bacteria	67 (57.3)
G + bacteria	31 (26.5)
Fungus	11 (9.4)
Others	18 (15.4)
Total culture negative	17 (14.5)
APACHE II score, mean ± SD	11.8 ± 5.7
SOFA score, mean ± SD	4.7 ± 1.8
SOFA score-Respiratory system, mean ± SD	1.2 ± 0.5
SOFA score-Nervous system, mean ± SD	0.5 ± 0.5
SOFA score-Cardiovascular system, mean ± SD	0.6 ± 0.6
SOFA score-Liver, mean ± SD	0.8 ± 0.6
SOFA score-Coagulation, mean ± SD	1.0 ± 0.5
SOFA score-Renal system, mean ± SD	0.7 ± 0.6
**Laboratory detections**	
TNF-α (pg/mL), median (IQR)	157.7 (115.5–246.5)
IL-1β (pg/mL), median (IQR)	7.4 (5.6–9.3)
IL-6 (pg/mL), median (IQR)	43.0 (32.9–52.8)
CRP (mg/L), median (IQR)	80.0 (38.4–122.4)

SD, standard deviation; BMI, body mass index; CKD, chronic kidney disease; CCVD, cardiovascular and cerebrovascular diseases; G-, Gram-negative; G+, Gram-positive; APACHE II, Acute Physiology and Chronic Health Evaluation II; SOFA, Sequential Organ Failure Assessment; TNF-α, tumor necrosis factor-alpha; IQR, interquartile range; IL-1β, interleukin-1beta; IL-6, interleukin 6; CRP, C-reactive protein.

### Association of miR-451a with Th1 cells, Th2 cells, and Th1/Th2 ratio

**FIGURE 1:**
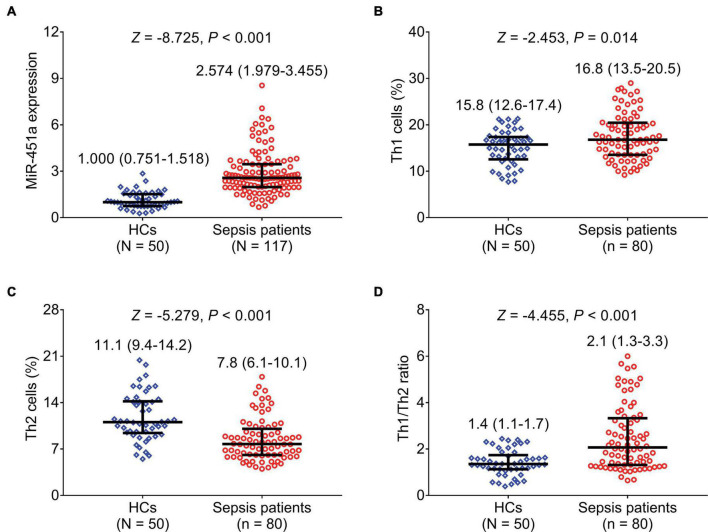
MiR-451a, Th1 cells, and Th2 cells were dysregulated in patients with sepsis. Comparison of miR-451a **(A)**, Th1 cells **(B)**, Th2 cells **(C)**, and the Th1/Th2 ratio **(D)** between patients with sepsis and HCs.

**FIGURE 2:**
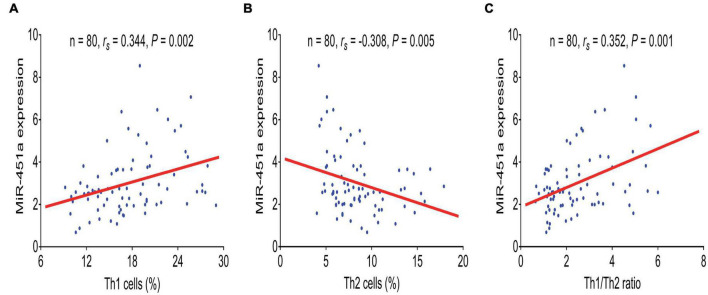
MiR-451a was positively correlated with Th1 cells and the Th1/Th2 ratio. Association of miR-451a with Th1 cells **(A)**, Th2 cells **(B)**, and the Th1/Th2 ratio **(C)** in patients with sepsis.

MiR-451a (*p* < 0.001), Th1 cells (*p* = 0.014), and the Th1/Th2 ratio (*p* < 0.001) were elevated, while Th2 cells (*p* < 0.001) were declined in the patients with sepsis compared to HCs (Figures 1A–D). Moreover, miR-451a was positively linked with Th1 cells (*r_*s*_* = 0.344, *p* = 0.002) (Figure 2A), while it was negatively related to Th2 cells (*r_*s*_* = –0.308, *p* = 0.005) (Figure 2B) in the patients with sepsis. Also, miR-451a was positively related to the Th1/Th2 ratio (*r_*s*_* = 0.352, *p* = 0.001) in the patients with sepsis (Figure 2C).

### Correlation of miR-451a and Th1/Th2 ratio with inflammatory cytokines and acute physiology and chronic health evaluation II score

**FIGURE 3:**
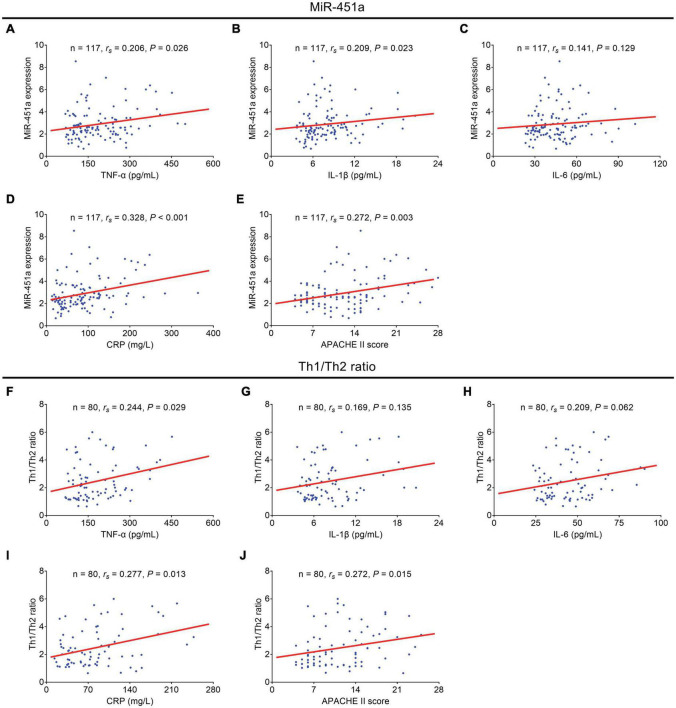
MiR-451a and the Th1/Th2 ratio were positively correlated with inflammatory cytokines and the APACHE II score. Correlation of miR-451a with TNF-α **(A)**, IL-1β **(B)**, IL-6 **(C)**, CRP **(D)**, and the APACHE II score **(E)** in patients with sepsis. Correlation of the Th1/Th2 ratio with TNF-α **(F)**, IL-1β **(G)**, IL-6 **(H)**, CRP **(I)**, and the APACHE II score **(J)** in patients with sepsis.

MiR-451a was positively associated with TNF-α (*r_*s*_* = 0.206, *p* = 0.026), IL-1β (*r_*s*_* = 0.209, *p* = 0.023), CRP (*r_*s*_* = 0.328, *p* < 0.001), and the APACHE II score (*r_*s*_* = 0.272, *p* = 0.003), while it did not relate to IL-6 (*r_*s*_* = 0.141, *p* = 0.129) in the patients with sepsis (Figures 3A–E). Meanwhile, the Th1/Th2 ratio was positively related to TNF-α (*r_*s*_* = 0.244, *p* = 0.029), CRP (*r_*s*_* = 0.277, *p* = 0.013), and the APACHE II score (*r_*s*_* = 0.272, *p* = 0.015), while no correlation of the Th1/Th2 ratio with IL-1β (*r_*s*_* = 0.169, *p* = 0.135) or IL-6 (*r_*s*_* = 0.209, *p* = 0.062) was observed in the patients with sepsis (Figures 3F–J).

### Association of miR-451a and Th1/Th2 ratio with sequential organ failure assessment score

MiR-451a (*r_*s*_* = 0.372, *p* < 0.001) and the Th1/Th2 ratio (*r_*s*_* = 0.265, *p* = 0.017) were positively related to the overall SOFA score in the patients with sepsis (Table 2). Also, miR-451a and the Th1/Th2 ratio were positively associated with some organ-specific SOFA scores in the patients with sepsis (such as SOFA score-respiratory system, SOFA score-cardiovascular system, SOFA score-liver, SOFA score-coagulation, SOFA score-renal system) (all *p* < 0.05).

**TABLE 2:** Correlation of miR-451a and the Th1/Th2 ratio with the SOFA score in patients with sepsis.

Items	MiR-451a (*n* = 117)		Th1/Th2 ratio (*n* = 80)	
		
	*r*_*s*_	*P*-value	*r*_*s*_	*P*-value
SOFA score	0.372	**<0.001**	0.265	**0.017**
SOFA score-respiratory system	0.291	**0.001**	0.259	**0.020**
SOFA score-nervous system	0.108	0.248	–0.103	0.364
SOFA score-cardiovascular system	0.243	**0.008**	–0.024	0.830
SOFA score-liver	0.219	**0.018**	0.316	**0.004**
SOFA score-coagulation	0.174	0.060	0.368	**0.001**
SOFA score-renal system	0.315	**0.001**	0.162	0.151

MiR-451a, microRNA-451a; Th1, T helper 1 cells; Th2, T helper 2 cells; SOFA, Sequential Organ Failure Assessment. The bold values represent statistical significance.

### Association of miR-451a, and Th1/Th2 ratio with mortality

**FIGURE 4:**
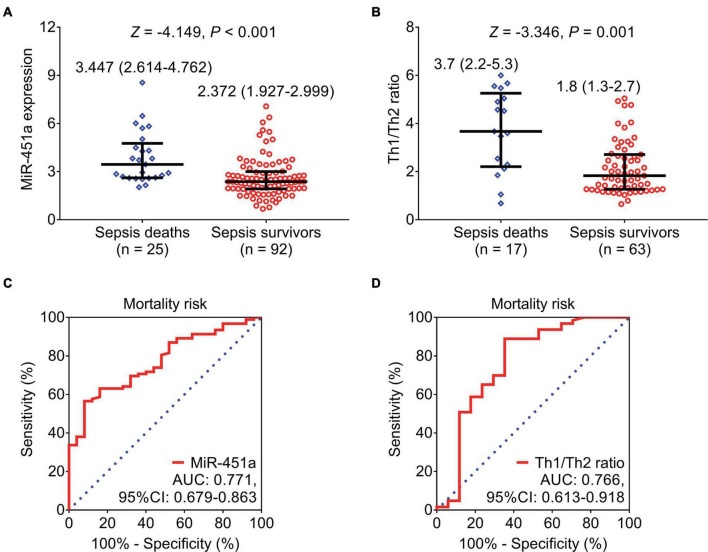
MiR-451a and the Th1/Th2 ratio could estimate mortality risk. Comparison of miR-451a **(A)** and the Th1/Th2 ratio **(B)** between sepsis survivors and sepsis deaths. ROC curve analyses of miR-451a **(C)** and the Th1/Th2 ratio **(D)** in estimating mortality risk in patients with sepsis.

MiR-451a (*p* < 0.001) (Figure 4A) and the Th1/Th2 ratio (*p* = 0.001) (Figure 4B) were both elevated in sepsis deaths compared to sepsis survivors. Furthermore, ROC curve analyses exhibited that both miR-451a [area under curve (AUC): 0.771, 95% confidence interval (CI): 0.679–0.863] (Figure 4C) and the Th1/Th2 ratio (AUC: 0.766, 95% CI: 0.613–0.918) (Figure 4D) could estimate mortality risk in patients with sepsis.

### Factors relating to mortality risk

Even though miR-451a [odds ratio (OR): 1.777, *p* < 0.001] the Th1/Th2 ratio (OR: 2.238, *p* < 0.001), Th1 cells (OR: 1.215, *p* = 0.002), and Th2 cells (OR: 0.786, *p* = 0.045) were related to 28-day mortality risk from univariate logistic regression analysis; only the Th1/Th2 ratio (OR: 2.052, *p* = 0.005) was independently associated with elevated 28-day mortality risk by multivariate logistic regression analysis (Table 3). Besides, G- bacteria (yes vs. no) (OR = 0.191, *p* = 0.037) and the SOFA score (OR = 1.867, *p* = 0.002) could serve as independent factors for estimating 28-day mortality risk in patients with sepsis.

**TABLE 3:** Factors relating to the 28-day mortality risk in patients with sepsis by logistic regression model analysis.

Items	*P*-value	OR	95%CI	
			
			Lower	Upper
**Univariate logistic regression**				
MiR-451a	**<0.001**	1.777	1.286	2.455
Th1/Th2 ratio	**<0.001**	2.238	1.455	3.441
Th1	**0.002**	1.215	1.076	1.373
Th2	**0.045**	0.786	0.620	0.995
Age	0.904	1.002	0.966	1.040
Gender (Male vs. Female)	0.590	1.307	0.493	3.462
BMI	0.251	1.072	0.952	1.206
Smoke (Yes vs. No)	0.222	0.677	0.362	1.266
Drink (Yes vs. No)	0.229	1.731	0.708	4.233
History of hypertension (Yes vs. No)	0.318	1.575	0.646	3.840
History of hyperlipidemia (Yes vs. No)	0.079	2.593	0.895	7.507
History of diabetes (Yes vs. No)	0.171	2.306	0.696	7.634
History of CKD (Yes vs. No)	0.912	0.913	0.181	4.598
History of CCVD (Yes vs. No)	0.627	1.298	0.453	3.719
**Primary infection site**				
Abdominal infection	Ref.			
Respiratory infection	0.057	3.167	0.964	10.398
Skin and soft tissue infection	0.317	2.000	0.515	7.764
Other infections	0.085	3.455	0.844	14.144
**Primary organism**				
G- bacteria (Yes vs. No)	**0.018**	0.330	0.131	0.827
G + bacteria (Yes vs. No)	0.750	0.846	0.303	2.362
Fungus (Yes vs. No)	0.051	3.583	0.994	12.923
Others (Yes vs. No)	0.261	0.413	0.088	1.931
Total culture negative (Yes vs. No)	**0.001**	5.906	1.981	17.605
APACHE II score	**0.004**	1.124	1.039	1.216
SOFA score	**<0.001**	2.019	1.501	2.715
SOFA score-respiratory system	**<0.001**	7.820	3.017	20.271
SOFA score-nervous system	0.392	1.456	0.616	3.439
SOFA score-cardiovascular system	**0.005**	3.263	1.421	7.495
SOFA score-liver	**0.011**	2.752	1.266	5.982
SOFA score-COAGULATION	**0.019**	2.875	1.192	6.937
SOFA score-renal system	**0.001**	4.102	1.779	9.459
TNF-α (pg/mL)	0.077	1.004	1.000	1.008
IL-1β (pg/mL)	0.263	1.069	0.951	1.202
IL-6 (pg/mL)	0.366	1.013	0.985	1.041
CRP (mg/L)	**0.001**	1.012	1.005	1.019
**Multivariate logistic regression (Step forward method)**				
Th1/Th2 ratio	**0.005**	2.052	1.243	3.386
G- bacteria (Yes vs. No)	**0.037**	0.191	0.040	0.908
SOFA score	**0.002**	1.867	1.265	2.756

OR, odds ratio; CI, confidence interval; MiR-451a, microRNA-451a; Th1, T helper 1 cells; Th2, T helper 2 cells; BMI, body mass index; CKD, chronic kidney disease; CCVD, cardiovascular and cerebrovascular diseases; G-, Gram-negative; G +, Gram-positive; APACHE II, Acute Physiology and Chronic Health Evaluation II; SOFA, Sequential Organ Failure Assessment; TNF-α, tumor necrosis factor-alpha; IQR, interquartile range; IL-1β, interleukin-1beta; IL-6, interleukin 6; CRP, C-reactive protein. The bold values represent statistical significance.

## Discussion

MiR-451a and the Th1/Th2 ratio have also been previously reported to be dysregulated in patients with sepsis (Xue et al., 2019, 2020; Wang et al., 2020; Yu et al., 2021). In particular, miR-451a and Th1 cells are upregulated, while Th2 cells are downregulated in the patients with sepsis (Xue et al., 2019, 2020; Wang et al., 2020; Yu et al., 2021). More importantly, miR-451a is able to regulate the differentiation of CD4^+^ T cells into Th cells, while the relation between miR-451a and Th cells in patients with sepsis remains unexplored (Cheng et al., 2017). In line with previous studies, the present study observed that miR-451a, Th1 cells, and the Th1/Th2 ratio were upregulated, while Th2 cells were downregulated in patients with sepsis compared to HCs, which could be explained as that miR-451a and Th1 cells were closely involved in the imbalanced inflammatory cytokines and immune dysfunction, which were the two hallmarks of sepsis; thus, miR-451a and Th1 cells were elevated in patients with sepsis compared to HCs (Cheng et al., 2017; Huang et al., 2019). Apart from that, we also discovered that miR-451a was positively related to Th1 cells and the Th1/Th2 ratio, while it was negatively related to Th2 cells in patients with sepsis. This could be explained: miR-451a promoted the differentiation of CD4^+^ T cells into Th1 cells; thus, a positive correlation of miR-451a with Th1 cells in patients with sepsis was shown (Cheng et al., 2017).

The association of miR-451a and Th cells with septic organ injury is also of great interest. One study observes that miR-451a is positively related to CRP and septic organ injury as shown by SOFA and APACHE II scores in patients with sepsis (Wang et al., 2020). Interestingly, one recent study has reported that Th1 cells are positively associated with the APACHE II score in patients with sepsis patients, but no correlation of Th1 cells with the SOFA score is observed (Yu et al., 2021). In the present study, it was observed that miR-451a and the Th1/Th2 ratio were both positively related to TNF-α, CRP, the APACHE II score, and the SOFA score in patients with sepsis, which could be explained as that: (a) miR-451a enhanced the inflammatory response through the regulation of multiple signaling pathways [such as nuclear factor (NF)-κB and mitogen-activated protein kinase (MAPK) pathways], thus relating to a positive association with inflammatory cytokines in patients with sepsis (Murata et al., 2014; Li et al., 2019). (b) Th1 cells were able to release proinflammatory cytokines, which further recruited more immune cells (such as neutrophils) and caused a systematic inflammation, thus leading to a positive relationship between Th1 cells and inflammation markers in patients with sepsis (Dong, 2021). (c) upregulated inflammatory cytokines contributed to cell apoptosis and macrophage autophagy, thus leading to organ damage and immune dysfunction, which was further associated with advanced septic organ injury in patients with sepsis (Cheng et al., 2016; Huang et al., 2019; Qiu et al., 2019).

MiR-451 and the Th1/Th2 ratio display certain prognostic values in estimating mortality in patients with sepsis. For instance, elevated miR-451a is related to septic shock occurrence and cardiac dysfunction in patients with sepsis (Wang et al., 2020). Also, Th1 cells are increased in septic deaths compared to sepsis survivors (Yu et al., 2021). Moreover, a higher Th1/Th2 ratio is associated with death in patients with sepsis (Xue et al., 2019). In line with these previous studies, it was discovered that elevated miR-451a and the Th1/Th2 ratio were associated with sepsis deaths; also, they could estimate mortality risk in patients with sepsis. The following explanations were applied: (a) miR-451a promoted systemic inflammation through regulating multiple pathways as indicated earlier, thus increasing the likelihood of developing excessive immune reactions and following complications (such as coagulation disorders and acute lung injury, etc.), therefore leading to elevated mortality risk in patients with sepsis (Huang et al., 2019; Ma et al., 2019; Kumar, 2020). (b) Th1 cells and their secreted cytokines could induce cellular oxidative stress to impair the intracellular mitochondria, which further caused cell apoptosis in multiple critical organs (such as the kidney, the liver, etc.) and led to multiple organ failure and mortality in patients with sepsis (Chavez and Tse, 2021). Moreover, miR-451a was related to elevated 28-day mortality risk in patients with sepsis, but it failed to serve as an independent factor. The possible reason to explain this finding was that miR-451a was closely related to the Th1/Th2 ratio as discussed earlier; thus, its prognostic value might be impaired by the Th1/Th2 ratio in multivariate logistic regression analysis; hence, miR-451a was not able to serve as one independent factor for estimating mortality risk in patients with sepsis (Cheng et al., 2017).

Although the current study focused on the clinical role of Th1 cells and Th2 cells in patients with sepsis, other Th subfamily members also exhibit certain diagnostic and prognostic values in sepsis. Taking Th17 cells as an example, higher Th17 cells and their secreted cytokines IL-17A are observed in patients with sepsis compared to HCs; also, they relate to advanced septic organ injury (Li et al., 2020; Yu et al., 2021). Moreover, Th17 cells are linked with elevated mortality risk in patients with sepsis (Zhao et al., 2021). Apart from Th17 cells and their secreted IL-17A, other cytokines also display critical clinical values in sepsis management. For instance, the upregulated interferon (IFN)-γ is related to a higher risk of developing fungal infection at a late stage in patients with early sepsis (Kim et al., 2020). Also, IL-2 and IFN-γ expressions are reported to be enhanced in the patients with sepsis (Guan et al., 2020). Given the huge amount and complex interaction among Th subfamily members, it is not ideal to detect all these Th subfamily members and their secreted cytokines in one study. Thus, a series of studies might be conducted to investigate the clinical application of various Th subfamily members in sepsis.

One thing that needs to be clarified is that all samples from 117 patients with sepsis are utilized for miR-451a detection by RT-qPCR, but only samples from 80 patients with sepsis are used for Th1/Th2 determination by flow cytometry. The reason for this unmatched number is due to the extensive clinical work in the hospital and the shortage in sorting, delivering, and analyzing samples from the inpatient ward to the laboratory. This may cause a prolonged time interval between sampling and analyzing (> 24 h), and further results in an invalided sample for Th1/Th2 detection using flow cytometry.

Several limitations were exhibited in the current study. For instance, the sample size was limited, and forthcoming studies with a larger sample size were desired to validate these findings. Moreover, multiple time-point detections of miR-451a in patients with sepsis were warranted in the following studies to observe its change during the disease course. Furthermore, the molecular experiment to discover the detailed mechanisms of miR-451a on regulating Th cells in sepsis was warranted.

## Conclusion

In conclusion, miR-451a correlates with the Th1/Th2 ratio, and they both relate to inflammation, septic organ injury, and mortality risk in patients with sepsis.

## Data availability statement

The original contributions presented in this study are included in the article/supplementary material, further inquiries can be directed to the corresponding author.

## Ethics statement

The studies involving human participants were reviewed and approved by the Ethics Committee of The Central Hospital of Wuhan, Tongji Medical College, Huazhong University of Science and Technology. The patients/participants provided their written informed consent to participate in this study.

## Author contributions

LY contributed to the conception and design. FG contributed to the data acquisition, data analysis, and interpretation of data. WL contributed to drafting and revising of the article. All authors contributed to the article and approved the final manuscript.
